# ACCURACY AND USABILITY OF A MOBILE ALERT SYSTEM FOR COMMUNITY CITIZENS TO LOCATE PERSONS WITH DEMENTIA WHO GET LOST

**DOI:** 10.1093/geroni/igz038.1698

**Published:** 2019-11-08

**Authors:** Christine H Daum, Noelannah Neubauer, Carlo Oliva, Ron Beleno, Lili Liu

**Affiliations:** 1 University of Alberta, Edmonton, Alberta, Canada; 2 Independent/Community ASAP, Toronto, Ontario, Canada

## Abstract

Three out of five persons with dementia wander. While the literature supports community engagement in the location of lost older adults, publically funded Silver Alert programs are associated with jurisdictional issues and alert fatigue. In collaboration and consultation with key stakeholders (older adults living with dementia, care partners, service providers, advocates, police organizations), we developed Community ASAP, an alert system (mobile website and app) that mobilizes community citizens who volunteer to keep watch for persons with dementia reported to be lost. The purpose of this study was to evaluate usability and functionality of the Community ASAP app. Thirteen participants from six regions in Canada received a total of 130 missing person notifications. They recorded the time and content of these notifications, completed the Website Usability Questionnaire, and provided written feedback or participated in a group interview about their experiences using the app and suggestions for improvements. The Community ASAP app delivered notifications with 100% accuracy and received messages from participants with 98% accuracy. Overall, participants thought the interface was easy to navigate, graphics were pleasing, easy to use, and was clearly organized. Suggestions for improvements to increase usability included: 1) Multi-sensory alerts to make them more noticeable and increase readability; 2) Clearer navigation within the home screen and preferences; 3) User support (instructions, demo video, technical support). Evaluation results for this innovative app were favourable; suggestions will be used to further improve usability, particularly for end users who are novices at using mobile applications.

